# Risk for Persistent Peripheral Neuropathy After Repair of Brachial Artery Injuries

**DOI:** 10.7759/cureus.22997

**Published:** 2022-03-09

**Authors:** Scott N Loewenstein, Corianne Rogers, Vasil V Kukushliev, Joshua Adkinson

**Affiliations:** 1 Plastic and Reconstructive Surgery, Medical College of Wisconsin, Milwaukee, USA; 2 Plastic and Reconstructive Surgery, Indiana University School of Medicine, Indianapolis, USA

**Keywords:** orthopaedic hand surgery, brachial artery transection, brachial, sensitive neuropathy, motor neuropathy

## Abstract

Background

Brachial artery lacerations are limb-threatening injuries requiring emergent repair. Concomitant peripheral nerve symptoms are often only identified postoperatively. This study evaluated the prevalence of peripheral nerve deficits among this population as the indications for early nerve exploration have not been definitively established.

Methods

We reviewed all patients sustaining a brachial artery injury at one pediatric and two adult Level I Trauma Centers between January 1, 2007, and December 31, 2017. We recorded patient demographics, comorbidities, intoxication status, injury mechanism, concomitant injuries, type of repair, and intraoperative peripheral nerve exploration findings. Pre-and post-operative and long-term peripheral nerve function examination findings were analyzed. Differences between categorical variables were determined with Chi-square and Fisher’s exact tests.

Results

Thirty-four patients sustained traumatic brachial artery lacerations requiring operative repair. Injury mechanisms included tidy (clean cut) laceration (n=11, 32%), gunshot wound (n=9, 26%), blunt trauma (n=8, 24%), and untidy laceration (n=6, 18%). Preoperatively, 15% had a normal peripheral nerve examination, 26% had localizable symptoms, 38% had non-localizable symptoms, and 21% were taken to the operating room without formal nerve assessment. Thirty-two percent underwent formal nerve exploration, and 81% underwent nerve repair. At an average follow-up of 2.5 years, 27% of patients underwent exploration, and 39% did not have localizable peripheral nerve deficits (*p*=0.705).

Conclusions

Brachial artery injuries are associated with a clinically significant risk for long-term peripheral nerve symptoms. Early nerve exploration in patients with peripheral nerve symptoms after a brachial artery injury may be warranted, although there is no statistically significant likelihood for improved peripheral neurological outcomes.

## Introduction

Upper extremity arterial injuries are relatively rare, ranging from 0.48% in civilian trauma admissions [1] to 0.68% in combat-related injuries [2]. In patients with isolated upper extremity trauma, the incidence is slightly higher, up to 3.3% [3,4]. These injuries most frequently occur after penetrating trauma (i.e., stabbing, glass laceration, and gunshot wounds), but also can occur with blunt trauma (i.e., after accidents including motor vehicle collisions, falls, and direct assault) [1,5]. When present, upper extremity arterial injuries are often limb-threatening and require immediate operative intervention. Because many of these patients are taken urgently to the operating room for vascular repair, concomitant nerve injuries may not be discovered until after arterial reconstruction.

Peripheral nerve deficits are found in 46%-62.1% of patients with traumatic brachial artery injuries [1,5-9]. Many of these deficits are neuropraxic lesions, whereas a smaller percentage sustain partial or complete neurotmetic lesions. Neuropraxic lesions may occur, for example, after a gunshot injury. A substantial transfer of energy occurs from a high-momentum bullet to the tissue adjacent to the peripheral nerve. Neuropraxia may also result from a displaced humerus fracture or a compressive hematoma from an adjacent vascular injury [8].

When neurotmesis is present, the prognosis for spontaneous nerve recovery is poor. Mechanisms commonly producing neurotmesis include laceration due to glass, a knife, or a bullet passing directly through the nerve fascicles. In these cases, meaningful return of function depends on time-dependent reinnervation of the muscle motor endplates. Denervated muscle progressively loses functionality of motor endplates with a half-life of 140 days. After 12-18 months, meaningful reinnervation is impossible [10]. Reinnervation may be improved with surgical exploration and repair. The prognosis for recovery after the repair is best for tidy lacerations; the worst prognosis occurs in a concomitant vascular injury [11]. Without successful reconstruction of a peripheral nerve injury, many patients are at risk for persistent pain and a decreased quality of life [12,13]. Further, loss of peripheral nerve function impairs one’s ability to perform simple, everyday tasks such as bathing, eating, and grooming. 

Among patients with brachial artery injuries, peripheral nerve palsies are frequently discovered only after the patient is awakened from anesthesia for the vascular repair. When this occurs, the peripheral nerve surgeon is left with a challenging clinical conundrum - should the patient be urgently taken back to the operating room for nerve exploration and repair, or should the deficit be monitored for spontaneous recovery? This study sought to identify the peripheral nerve function outcomes in patients requiring brachial artery reconstruction for trauma with or without simultaneous peripheral nerve exploration.

## Materials and methods

After receiving approval from the Indiana University Institutional Review Board (Approval number: 1806913560), a retrospective chart review was performed for all patients sustaining traumatic brachial artery injuries at one pediatric and two adult urban midwestern Level I Trauma Centers between January 1, 2007, and December 31, 2017. Under the direction of the research team, a data analyst at the Regenstreif institute screened for eligible patients using the Indiana Network for Patient Care (INPC) health-information exchange, based on International Classification of Disease (ICD)-9 code 903.1 or ICD-10 code S45.1 combined with Current Procedural Terminology (CPT®) Codes 35206, 35236, or 35266. This query produced a list of encounters that two physicians subsequently reviewed in parallel (S.L and C.R.) to build a de-identified database of patients with traumatic brachial artery injuries requiring surgical repair. We then recorded patient demographics, comorbidities, alcohol, and drug intoxication status, injury mechanism, concomitant injuries, preoperative peripheral nerve function examination findings, type of peripheral nerve repair, intraoperative peripheral nerve exploration findings, short and long-term post-operative peripheral nerve function examination findings, and mean follow-up. Short-term nerve examination findings were defined as less than or equal to 7 days from surgery. The long-term peripheral nerve function examination findings were considered the last peripheral nerve function examination (greater than seven days from surgery). Univariate analysis was performed using Microsoft Excel (Redmond, WA) to calculate descriptive statistics. The Fisher exact test was used to examine for differences between proportions of patients with peripheral nerve deficits.

## Results

Thirty-four patients sustained traumatic brachial artery injuries requiring operative repair during the study period. Patient characteristics are presented in Table 1. The average patient age was 25.5 years (standard deviation 12.9 years).

**Table 1:** Patient characteristics.

Patient characteristics	n (%)
Sex	
Male	28 (82.4)
Female	6 (17.6)
Race	
White	21 (61.8)
Black	12 (35.3)
American Indian or Alaskan Native	0 (0.0)
Asian	0 (0.0)
Native Hawaiian and Other Pacific Islander	0 (0.0)
Two or more races	0 (0.0)
Not provided	1 (2.9)
Comorbidities	
Tobacco abuse	15 (44.1)
Diabetes	0 (0.0)
Peripheral vascular disease	0 (0.0)
Renal disease	0 (0.0)
Chronic obstructive pulmonary disease	0 (0.0)
Depression	2 (5.9)
Anxiety	2 (5.9)
Other psychiatric comorbidities	6 (17.6)

Injury characteristics are presented in Table 2. The brachial artery injury was treated by means of an interposition vein graft in 24 (71%), primary repair in 6 (18%), patch angioplasty in 3 (9%), and by undocumented means in 1 (3%). Upper extremity fasciotomies were performed in 6 patients (18%).

**Table 2:** Injury characteristics. n = number; OR, operating room

Injury Characteristics	n (%)
Mechanism	
Gunshot wound	9 (26.5)
Motor vehicle collision	2 (5.9)
Tidy (clean cut) laceration (e.g., knife, glass)	11 (32.4)
Multiple cuts, untidy wound	6 (17.6)
Crush	0 (0.0)
Fall	6 (17.6)
Involved extremity	
Right	19 (55.9)
Left	15 (44.1)
Distant organ system involved	8 (23.5)
Associated injury distal to elbow	5 (14.7)
Associated injury proximal to shoulder	3 (8.8)
Associated ipsilateral humerus fracture	13 (38.2)
Glasgow Coma Scale Score <15	7 (20.6)
Intubated prior to OR	4 (11.8)
Ethanol intoxication	
Tested positive	10 (29.4)
Tested negative	16 (47.1)
Not tested	8 (23.5)
Drug intoxication	
Tested positive	4 (11.8)
Tested negative	10 (29.4)
Not tested	20 (58.8)

Eleven patients underwent formal, concurrent peripheral nerve exploration by a peripheral nerve specialist. Eight patients (73%) identified nerve injuries primarily repaired; 1 underwent cable nerve grafting, and 2 underwent exploration alone. 

The pre-and post-operative and at-last follow-up peripheral nerve function examinations are presented in Table 3. Twenty-two patients had a documented peripheral nerve function examination deficit preoperatively, 19 had abnormalities immediately postoperatively, and 12 had abnormalities at last follow-up. At an average follow-up of 2.5 years (range 0-10.5 years), there was no statistically significant difference in the likelihood for persistent peripheral nerve function deficits in patients who did not undergo nerve exploration (39%) compared to patients who did undergo exploration (27%, p=0.705). 

**Table 3:** Neurological deficits. Preop = Preoperative; Postop = Postoperative

	Nerve Exploration				No Nerve Exploration		
Nerve	Preop	Postop	Last follow-up		Preop	Postop	Last follow-up
	n (%)	n (%)	n (%)		n (%)	n (%)	n (%)
Radial	0 (0.0)	1 (9.1)	1 (9.1)		1 (4.3)	1 (4.3)	0 (0.0)
Ulnar	1 (9.1)	0 (0.0)	1 (9.1)		3 (13.0)	3 (13.0)	3 (13.0)
Median	0 (0.0)	2 (18.2)	0 (0.0)		2 (8.7)	4 (17.4)	1 (4.3)
Musculocutaneous	0 (0.0)	0 (0.0)	0 (0.0)		1 (4.3)	0 (0.0)	1 (4.3)
Non-terminal branch	4 (36.4)	4 (36.4)	1 (9.1)		10 (43.5)	4 (17.4)	4 (17.4)
Normal examination	1 (9.1)	1 (9.1)	2 (18.2)		4 (17.4)	5 (21.7)	11 (47.8)
No data	5 (45.5)	3 (27.3)	6 (54.5)		2 (8.7)	6 (26.1)	3 (13.0)

## Discussion

This study reviewed peripheral nerve function examination outcomes for patients who underwent brachial artery repair for a traumatic injury. We found that peripheral nerve function deficits may persist in a substantial number of patients, regardless of concomitant peripheral nerve exploration (27% if nerve explored/repaired versus 39% if not explored, p=0.705). Peripheral nerve function deficits associated with brachial artery injuries are often complex high nerve injuries [5], where the prognosis for nerve function is directly related to the time to repair [14]. Early repair theoretically offers the best chance for nerve recovery; however, the diagnostic dilemma occurs when differentiating neuropraxic with their chance for spontaneous recovery from neurotmetic injuries, which have a limited or no change for spontaneous recovery.

The decision to pursue early peripheral nerve exploration may be difficult, mainly if the neurological deficits are not discovered until after the patient has awakened from anesthesia for vascular repair. In our experience, this scenario is not uncommon since patients are urgently taken to the operating room to minimize ischemia time in the threatened, dysvascular limb. Suppose the peripheral nerve surgeon is consulted at or before the time of brachial artery repair. In that case, the decision for immediate peripheral nerve exploration is straightforward, as this can be completed with minimal additional morbidity under the same anesthetic administered for the arterial repair. However, for neurological deficits manifesting after brachial artery repair, returning to the operating room requires repeat surgical site exposure in a new arterial reconstruction setting, which may be associated with significant risk. Thus, avoiding a procedure in a patient without reparable injury is desirable. Given this clinical reality, patients with peripheral nerve function deficits after brachial artery repair can be counseled either for early return to the operating room for intraoperative nerve exploration or observation with serial clinical examinations and electrodiagnostic studies.

Evidence to support a particular treatment algorithm for peripheral nerve function deficits in the setting of a brachial artery repair is limited, likely related to the relative rarity of such injuries. Several authors acknowledge the high incidence of peripheral nerve injury symptoms among patients with brachial artery injuries but do not describe long-term peripheral nerve function examination outcomes [5,7]. Bercik et al. identified three patients with concomitant brachial artery and peripheral nerve deficits among patients with humerus fractures related to gunshot wounds. They found that the two required an exploration and treatment of a neuroma-in-continuity found due to lack of spontaneous recovery by 90 days [15]. Visser et al. reviewed their experience of 16 brachial artery injuries associated with nine peripheral nerve injuries at a single academic center from 1963-1978. Five underwent peripheral nerve repairs. Of these five patients, four had either no motor or sensory function (2 patients) or partial return of both motor and sensory function (2 patients and 1 of these 2 had plastic surgery to facilitate the partial motor return). One patient had complete motor and sensory function return at long-term follow-up [6]. Shaw et al. found that 4 of 8 patients requiring immediate operative repair for peripheral nerve penetrating injuries associated with brachial artery injury had a “good” recovery. However, they did not discuss the long-term clinical outcomes of those not explored [9]. In a similar cohort, Stanec et al. reported a 44.8% likelihood for functional recovery after a 5-6 month post-injury delay of exploration and nerve repair [16]. These studies indicate a poor functional outcome regardless of peripheral nerve exploration. In general, repair of peripheral nerves after arterial laceration has been met with limited success [8]. Our data also indicates that the prognosis for nerve recovery is poor and only slightly improved with early peripheral nerve exploration. 

**Figure 1:**
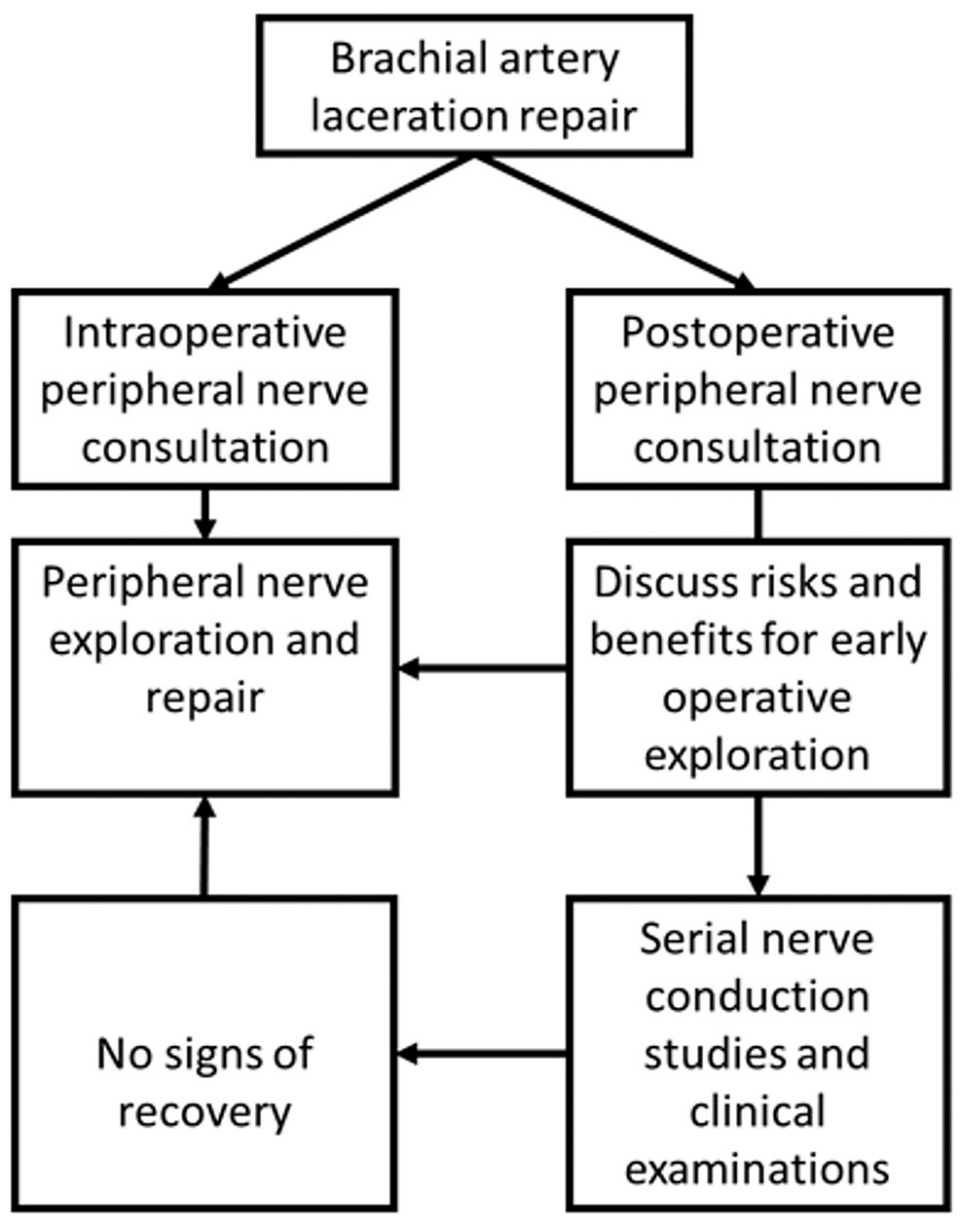
Algorithm for peripheral nerve function deficits associated with brachial artery injury.

Alternatively, early exploration and repair of a peripheral nerve injury may be the only chance for improved functional recovery. In this population, even a slight improvement may be considered clinically significant. This treatment approach is supported by the fact that the best chance for recovery in the event of a neurotmetic injury is the precise coaptation of proximal and distal nerve ends [17]. If the nerve exploration reveals an intact, uninjured nerve, the wound is closed, and the patient is monitored for nerve recovery. However, some percentage of this cohort will develop a neuroma-in-continuity, so functional improvement may remain compromised. If the early nerve exploration demonstrates a clear injury, it can be repaired with the goal of increasing the likelihood for axonal regrowth to distal targets prior to loss of all motor endplates. The potentially devastating loss of function with peripheral injuries [12,13] may reasonably outweigh the risks of nerve exploration. An algorithm to counsel patients with peripheral nerve function deficits after brachial artery repair for trauma is presented in Figure 1.

This study has several limitations. As a retrospective chart review, we cannot directly ascribe patient outcomes to the peripheral nerve intervention. Further, significant preoperative peripheral nerve examination findings were missing in several patients. This finding is not unexpected as many patients with a dysvascular extremity are urgently taken to surgery prior to formal nerve evaluation. Postoperatively, patients were divided into short-term (less than or equal to 7 days) and long-term (last follow-up more significant than seven days). Several patients were discharged after surgery and did not undergo a repeat peripheral nerve function examination until seven days. Their last follow-up was less than seven days from surgery for several other patients. The dropout effects are mitigated by using a health information exchange that included over 95% of acute inpatient and non-office-based outpatient clinical care provided in the metropolitan statistical area of the study population since 2004 [18]. Thus, patients with persistent peripheral nerve deficits that changed providers would likely be captured in the dataset. It is also possible that patients with improved peripheral nerve function did not seek further treatment; this may explain the higher loss to follow-up rate in the patients who did nerve exploration. Finally, there was no standardized outcomes assessment, such as Medical Research Council Scale grades, two-point discrimination, or Semmes-Weinstein monofilament ratings.

## Conclusions

This study reports the relatively high incidence of persistent peripheral nerve deficits among patients with traumatic brachial artery injuries that require operative repair. There is a higher likelihood for improved outcomes with early peripheral nerve intervention, although this difference was not statistically significant. As such, early nerve exploration in patients with peripheral nerve symptoms of dysfunction after a brachial artery injury may be warranted to improve long-term outcomes, but a more extensive, prospective study is recommended to provide definitive recommendations for treatment of this challenging problem. The data presented in this paper will facilitate a more informed discussion between surgeon and patient regarding treatment options and the possible role for early nerve exploration.
